# Adénocarcinome pulmonaire primitif: expérience d'un centre hospitalier tunisien

**DOI:** 10.11604/pamj.2015.21.216.6795

**Published:** 2015-07-24

**Authors:** Samah Joobeur, Hadhami Mribah, Ahmed Ben Saad, Saoussen Cheikh Mhamed, Houda Mahou, Naceur Rouatbi, Ali El Kamel

**Affiliations:** 1 Service de Pneumologie et d'Allergologie, Centre Hospitalo-Universitaire Fattouma Bourguiba de Monastir, Monastir 5000, Tunisie

**Keywords:** Adénocarcinome pulmonaire primitif, profil, survie, primary pulmonary adenocarcinoma, profile, survival

## Abstract

La fréquence de l'adénocarcinome pulmonaire primitif est en nette augmentation au dépend des autres types histologiques de cancer bronchique primitif. En effet, il représente environ 40% des cas des carcinomes bronchiques non à petites cellules (CNPC). Il se distingue par certaines particularités. Décrire les aspects épidémiologiques, cliniques, thérapeutiques et évolutifs de l'adénocarcinome pulmonaire primitif. Etude rétrospective incluant 322 patients porteurs d'adénocarcinome pulmonaire primitif, hospitalisés au service de pneumologie du centre hospitalo-universitaire de Monastir (Tunisie) entre janvier 1990 et septembre 2013. L’âge moyen de nos patients était de 59,4 ans. 25,8% sont âgés de moins de 50 ans. Une prédominance masculine (86,3%) a été notée. 81,7% des patients étaient tabagiques. La symptomatologie respiratoire était dominée par la douleur thoracique (57,1%) et la toux (46%). Au moment du diagnostic, 73,3% des patients étaient au stade métastatique. Les localisations secondaires les plus fréquentes étaient le poumon controlatéral (25,5%), la plèvre (21,1%) et l'os (19,25%). La prise en charge thérapeutique s'est basée essentiellement sur la chimiothérapie (48,5% des cas). Seulement 10,3% des patients ont bénéficié d'un traitement chirurgical. La médiane de survie de nos patients était de 6 mois avec une survie à 1 an, 3 ans et 5 ans respectivement de 25,9%, 3,2% et 2%. L'adénocarcinome bronchique primitif est un sous type histologique particulier parmi les cancers broncho-pulmonaires primitifs. Son incidence est en augmentation depuis une vingtaine d'année. Malgré les progrès thérapeutiques, il reste de mauvais pronostic.

## Introduction

Le cancer broncho-pulmonaire constitue un problème majeur de santé publique vu sa prévalence élevée et son pronostic sombre. Il occupe largement la première place parmi les tumeurs malignes de l'homme. L'adénocarcinome pulmonaire primitif est un type histologique particulier parmi les carcinomes non à petites cellules (CNPC). L'adénocarcinome primitif représente actuellement, le type histologique le plus fréquent des cancers broncho-pulmonaires primitifs. Son incidence est en augmentation depuis une vingtaine d'années [1, 2]. En Tunisie, on assiste actuellement à une augmentation de l'incidence de l'adénocarcinome atteignant 40% des tumeurs broncho-pulmonaires et représentant ainsi le type histologique le plus fréquent [3]. Le tabagisme n'est probablement pas le seul facteur responsable de l'augmentation de l'incidence de l'adénocarcinome bronchique. En effet, prés de 20% des patients ayant un adénocarcinome ne sont pas des fumeurs [4]. Ceci suppose l'existence d'autres facteurs nutritionnels, professionnels, environnementaux et génétiques qui pourraient expliquer cette augmentation [1, 5]. Malgré les progrès thérapeutiques, le pronostic de l'adénocarcinome bronchique reste réservé.

## Méthodes

Nous avons mené une étude rétrospective portant sur les dossiers des patients porteurs d'un adénocarcinome primitif du poumon pris en charge au service de pneumologie et d'allergologie au centre hospitalo-universitaire de Monastir (Tunisie) entre janvier 1990 et septembre 2013. Le but de cette étude est d'analyser les caractéristiques démographiques, cliniques, thérapeutiques et évolutives de l'adénocarcinome pulmonaire primitif dans notre région.

**Critères d'inclusion**: L'origine pulmonaire primitive a été retenue par une étude immuno-histochimique compatible (29,8%) ou la négativité du bilan de recherche d'un autre cancer primitif (selon le sexe et les éventuels signes d'appel) avec une présentation clinique, radiologique et endoscopique compatible avec un cancer primitif pulmonaire.

**Analyse statistique:** Les données collectées ont été saisies et analysées par le logiciel SPSS 17.0. Les variables quantitatives ont été exprimées en moyennes ± déviations standard (DS). Les variables qualitatives ont été exprimées en pourcentages. La survie a été analysée par la méthode de Kaplan Meier.

## Résultats

L’âge moyen de nos patients était de 59,4 ans avec des extrêmes allant de 23 à 94 ans. 25,8% des patients avaient un âge inférieur à 50 ans. Une nette prédominance masculine (86,3%) a été notée. 81,7% des patients sont tabagiques dont une femme avec une consommation moyenne de 47,1 PA. La majorité de nos malades était de grands fumeurs avec une consommation supérieure ou égale à 20 PA dans 92,4% des cas. Les antécédents pathologiques des patients étaient dominés par la BPCO (35%). Une histoire familiale de cancer a été notée dans 21 cas (6,4%), dont 13 cas (4%) de cancer bronchique primitif. Le délai moyen de consultation était de 2,43 mois, il était supérieur ou égal à 6 mois dans 7,6% des cas. Les signes fonctionnels respiratoires sont dominés par la douleur thoracique, la toux, la dyspnée et l'hémoptysie retrouvées respectivement dans 57,1%, 46%, 37,3% et 23,9% des cas. L'altération de l’état général était le signe extra-respiratoire le plus fréquemment retrouvé (59,3%). Le score de performance status de l'OMS utilisé pour l’évaluation de l’état général était supérieur ou égal à 2 dans 32,6% des cas au moment du diagnostic. L'examen physique a noté la présence d'un hippocratisme digital dans 13,6% des cas, d'un syndrome pleurétique dans 19% des cas, des adénopathies périphériques dans 13,3% des cas et un syndrome cave supérieur dans 3,7% des cas. La radiographie du thorax était pathologique dans 99,7% des cas, montrant des opacités intra-parenchymateuses dans 50,3% des cas, hilaires dans 29,2% des cas et médiastino-pulmonaires dans 20,2% des cas (Tableau 1). La fibroscopie bronchique a été réalisée chez 81,1% des patients. Elle était pathologique dans 70,5% des cas, révélant essentiellement une sténose bronchique, une infiltration de la muqueuse et un bourgeon endoluminal dans respectivement 32,6%, 51,6% et 17,9% des cas.

**Tableau 1:** Caractéristiques cliniques des patients

Caractéristiques	Moyenne ± DS	Nombre (%)
Age moyen	59,4 ± 11,5 ans	
Age moins de 50 ans		83 (25, 8%)
Sexe masculin		278 (86, 3%)
Tabagisme	47,1 ± 24,5 PA	263 (81, 7%)
BPCO		113 (35%)
Néoplasie dans la famille		21 (6, 4%)
Délai de consultation	2,43 ± 2,3 mois	
Symptomatologie clinique		
- douleur thoracique		184 (57, 1%)
- toux		148 (47%)
- hémoptysie		77 (23, 9%)
- altération de l’état général		191 (59, 3%)
Score PS de l'OMS ≥ 2		105 (32, 6%)
Hippocratisme digital		44 (13, 6%)
Adénopathies périphériques		43 (13, 3%)
Opacité radiologique intraparenchymateuse		162 (50, 3%)

**Figure 1:**
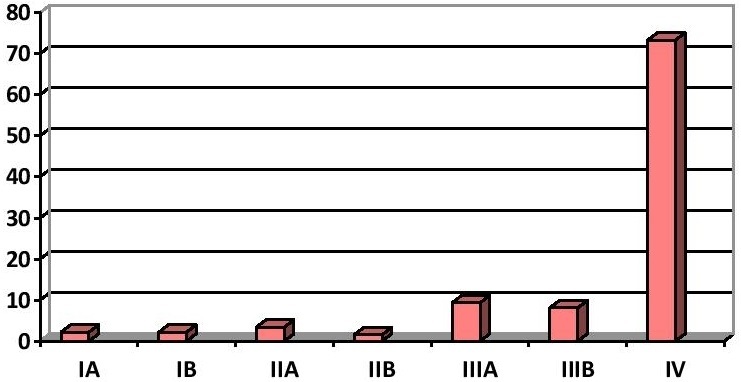
Stades TNM de l'adénocarcinome pulmonaire

La confirmation anatomo-pathologique a été obtenue par biopsie bronchique dans 36% des cas, par ponction transpariétale thoracique sous contrôle tomodensitométrique dans 24,2% des cas, par biopsie de métastases dans 24,3% des cas et par biopsie chirurgicale dans 7,3% des cas. Chez 8,4% des patients la confirmation du diagnostic était cytologique. L'origine pulmonaire primitive a été retenue par une étude immuno-histochimique compatible pratiquée chez 96 patients (29,8%) ou la négativité du bilan de recherche d'un autre cancer primitif avec une présentation clinique, radiologique et endoscopique compatible avec un cancer primitif pulmonaire chez les autres patients. Les profils immunohistochimiques les plus observés chez nos patients étaient CK7 positif (100% des cas), CK20 négatif (100% des cas) et TTF1 positif (64,6% des cas). Le bilan d'extension a conclu à un adénocarcinome localement avancé ou métastatique (stades IIIB et IV) dans 81,4% des cas (Figure 1). Les sites métastatiques les plus fréquemment observés étaient le poumon controlatéral (25,5% des cas), la plèvre (21,1% des cas), l'os (19, 3% des cas), les surrénales (17,1% des cas) suivis par le cerveau (16,15% des cas) et le foie (12,7% des cas). Sur le plan thérapeutique, seulement 10,3% des patients ont bénéficié d'un traitement chirurgical. Cette chirurgie était associée à un traitement adjuvant à base de radiothérapie et/ou chimiothérapie dans 66,6% des cas opérés. 9% des patients ont bénéficié de radiothérapie thoracique curative. La chimiothérapie a été indiquée dans 48,5% des cas. Elle était adjuvante dans 12,8% des cas. Le nombre moyen des cures était de 3,5. Des effets indésirables de la chimiothérapie sont apparus dans 83,3% des cas, dominés par les troubles ioniques et la toxicité hématologique. Une réponse objective était observée dans 34,7% des cas. 12,8% des patients ont bénéficié d'une chimiothérapie de 2ème ligne. 104 des patients (32,3%) ont bénéficié d'un simple traitement symptomatique pour une altération profonde de l’état général ou contre indication au traitement spécifique. Sur le plan évolutif la médiane de survie globale était de 6 mois avec une survie à 1an, 3 ans et à 5 ans de 25,9%, 3,2% et 2% respectivement.

## Discussion

La distribution des différents types histologiques du cancer bronchique primitif a nettement changé ces dernières années marquée par une incidence de plus en plus élevée de l'adénocarcinome aux dépens d'un déclin de celle du carcinome épidermoïde [1, 5, 6]. Ceci pourrait être expliqué par les variations des habitudes tabagiques et des composants des cigarettes [6–8]. De même, l'amélioration des techniques diagnostiques pour les tumeurs périphériques qui sont souvent des adénocarcinomes et les progrès importants des techniques anatomopathologiques faisant intégrer les données immunohistochimiques pourraient expliquer aussi l'augmentation de l'incidence de ce type de cancer. Dans notre étude, la fréquence de l'adénocarcinome parmi les CNPC diagnostiqués durant la même période d’étude (1990-2013) était de 46,5%. La revue de la littérature trouve que parmi les cas de CNPC, les patients ayant un adénocarcinome sont généralement moins âgés que ceux ayant un carcinome épidermoïde [9, 10]. L’âge moyen de notre population était de 59,4 ans. 25,8% des cas avaient un âge inférieur à 50 ans. De point de vue sexe, l'adénocarcinome est le type histologique prédominant chez la femme [5, 7]. Outre le tabagisme et la susceptibilité plus importante aux carcinogènes du tabac, d'autres facteurs peuvent expliquer la fréquence plus élevée de l'adénocarcinome dans cette population. Il s'agit essentiellement de la fréquence plus importante de la mutation de l'EGFR (epidermal growth factor) chez les femmes et l'importance de l'impact des facteurs hormonaux (estrogènes, progestérone) dans la stimulation de la carcinogenèse [11, 12]. Concernant l'approche histopathologique, l'adénocarcinome bronchique est défini histologiquement par la présence d'une différenciation glandulaire ou des signes de production de mucine avec une architecture variable. L’émergence de l'IHC a permis de distinguer avec une bonne sécurité entre origine pulmonaire primitive ou secondaire de l'adénocarcinome et d'apporter en plus des éléments pronostiques (marqueurs de prolifération, marqueurs de différenciation) ou prédictifs de la réponse au traitement (chimiothérapie ou thérapeutiques ciblées) [13]. Les profils immunohistochimiques les plus observés chez nos patients étaient CK7 positif/CK20 négatif. Le TTF1 était positif chez 64,6% des patients. L'adénocarcinome pulmonaire est souvent découvert à un stade avancé. En effet la majorité des auteurs rapportent une prédominance des stades localement avancés et métastatiques. Ceci est attribué en partie au retard de consultation des malades et parfois même au retard du diagnostic par les médecins en rapport avec l'absence de spécificité des symptômes [14, 15]. Sur le plan thérapeutique, des avancées thérapeutiques ces dernières années ont modifié considérablement la prise en charge des patients atteints de CNPC. Dans les stades précoces, le traitement de l'adénocarcinome rejoint celui des CNPC à quelques différences près. Dans les stades localement avancé et métastatique la prise en charge de ce type de cancer est révolutionnée par l'introduction de nouvelles drogues et aux progrès acquis dans le domaine de la chimiothérapie, ainsi que l'avènement des thérapies ciblées [16]. Ces dernières ne sont pas encore commercialisées dans notre pays. Malgré les progrès dans les modalités du diagnostic et du traitement de l'adénocarcinome bronchique primitif ces dernières années, le pronostic reste réservé. La survie varie selon les études et en fonction des critères d'inclusion [17, 18]. Dans notre série, la médiane de survie des malades porteurs d'adénocarcinome bronchique primitif était de 6 mois et la survie à 5 ans était de 2%.

## Conclusion

L'adénocarcinome pulmonaire primitif est un sous type histologique particulier parmi les cancers broncho-pulmonaires primitifs. Son incidence est en augmentation depuis une vingtaine d'année. Cette augmentation peut être attribuée à plusieurs facteurs: modifications des habitudes tabagiques, l'exposition au tabagisme passif, l'existence d'autres facteurs de risque (nutritionnels, professionnels, environnementaux et génétiques) et les progrès des techniques anatomopathologiques. Malgré les progrès thérapeutiques, il reste de mauvais pronostic. En l'absence de traitement réellement efficace, la meilleure stratégie actuelle reste la prévention par le renforcement des programmes de lutte anti-tabac et d'aide au sevrage tabagique.
